# Dysregulation of dopamine metabolism in children with spinal muscular atrophy type 1

**DOI:** 10.1093/braincomms/fcag402

**Published:** 2026-10-29

**Authors:** Tommaso Nuzzo, Barbara Risi, Valentina Bassareo, Adele D’Amico, Alberto Imarisio, Antonella Longo, Manolo Carta, Chiara Panicucci, Claudio Bruno, Enza Maria Valente, Massimiliano Filosto, Enrico Bertini, Francesco Errico, Alessandro Usiello

**Affiliations:** Laboratory of Translational Neuroscience, Ceinge Biotecnologie Avanzate ‘Franco Salvatore’, Naples 80145, Italy; Department of Environmental, Biological and Pharmaceutical Science and Technologies, Università degli Studi della Campania ‘Luigi Vanvitelli’, Caserta 81100, Italy; Department of Molecular and Translational Medicine, University of Brescia, Brescia 25123, Italy; NeMO-Brescia Clinical Center for Neuromuscular Diseases, Brescia 25064, Italy; Department of Biomedical Sciences, University of Cagliari, Monserrato 09042, Italy; Unit of Neuromuscular and Neurodegenerative Disorders, Department of Neurosciences, Bambino Gesù Children's Hospital IRCCS, Rome 00163, Italy; Department of Molecular Medicine, University of Pavia, Pavia 27100, Italy; Neurogenetics Research Centre, IRCCS Mondino Foundation, Pavia 27100, Italy; Unit of Neuromuscular and Neurodegenerative Disorders, Department of Neurosciences, Bambino Gesù Children's Hospital IRCCS, Rome 00163, Italy; Department of Biomedical Sciences, University of Cagliari, Monserrato 09042, Italy; Center of Translational and Experimental Myology, IRCCS Istituto Giannina Gaslini, Genoa 16147, Italy; Center of Translational and Experimental Myology, IRCCS Istituto Giannina Gaslini, Genoa 16147, Italy; Department of Neuroscience, Rehabilitation, Ophthalmology, Genetics, Maternal, and Child Health—DINOGMI, University of Genova, Genoa 16132, Italy; Department of Molecular Medicine, University of Pavia, Pavia 27100, Italy; Neurogenetics Research Centre, IRCCS Mondino Foundation, Pavia 27100, Italy; NeMO-Brescia Clinical Center for Neuromuscular Diseases, Brescia 25064, Italy; Department of Clinical and Experimental Sciences, University of Brescia, Brescia 25123, Italy; Unit of Neuromuscular and Neurodegenerative Disorders, Department of Neurosciences, Bambino Gesù Children's Hospital IRCCS, Rome 00163, Italy; Laboratory of Translational Neuroscience, Ceinge Biotecnologie Avanzate ‘Franco Salvatore’, Naples 80145, Italy; Department of Agricultural Sciences, University of Naples ‘Federico II’, Portici 80055, Italy; Laboratory of Translational Neuroscience, Ceinge Biotecnologie Avanzate ‘Franco Salvatore’, Naples 80145, Italy; Department of Environmental, Biological and Pharmaceutical Science and Technologies, Università degli Studi della Campania ‘Luigi Vanvitelli’, Caserta 81100, Italy

**Keywords:** spinal muscular atrophy, Nusinersen, motor function, dopamine metabolism

## Abstract

Spinal muscular atrophy (SMA) is a severe neuromuscular disorder caused by reduced expression of the survival motor neuron (SMN) protein. In addition to affecting motor neuron survival, SMN deficiency impacts multisystem physiology and neurotransmission. Dopaminergic dysfunction has been reported in mouse models of SMA, leading to postural and locomotor impairments that improve following treatment with levodopa and benserazide, which increase central dopamine availability. However, whether altered dopamine metabolism contributes to clinical symptoms in SMA patients remains unclear. To investigate this issue, we conducted a real-world observational study involving paediatric patients with SMA type 1 (SMA1), SMA2, and SMA3, and healthy controls. We quantified the main dopamine-related catabolites—3,4-dihydroxyphenylacetic acid (DOPAC) and homovanillic acid (HVA)—in cerebrospinal fluid samples collected at baseline and after five intrathecal doses of Nusinersen, an SMN-enhancing therapy. No significant differences were observed in DOPAC and HVA levels across SMA types and controls or following treatment, and no association emerged with *SMN2* copy number. In contrast, lower baseline DOPAC levels were detected in SMA1 patients requiring gastrostomy or tracheostomy, and were associated with reduced improvement following Nusinersen, as measured by changes in the Children's Hospital of Philadelphia Infant Test of Neuromuscular Disorders scores. Additional high-performance liquid chromatography analyses performed in an independent study cohort revealed significantly lower serum HVA levels in SMA1 patients compared to controls. In conclusion, our exploratory study suggests that altered dopaminergic metabolism is associated with greater disease severity and poorer motor outcomes following treatment, supporting an involvement of dopamine neurotransmission in clinical heterogeneity in SMA.

## Teaser

Nuzzo *et al*. report that cerebrospinal fluid dopamine metabolites do not differ across spinal muscular atrophy (SMA) types or after Nusinersen treatment. However, lower baseline 3,4-dihydroxyphenylacetic acid levels correlate with greater disease severity and poorer motor improvement in SMA type 1, suggesting altered dopaminergic metabolism in advanced disease.

## Graphical Abstract

**Graphical Abstract:**
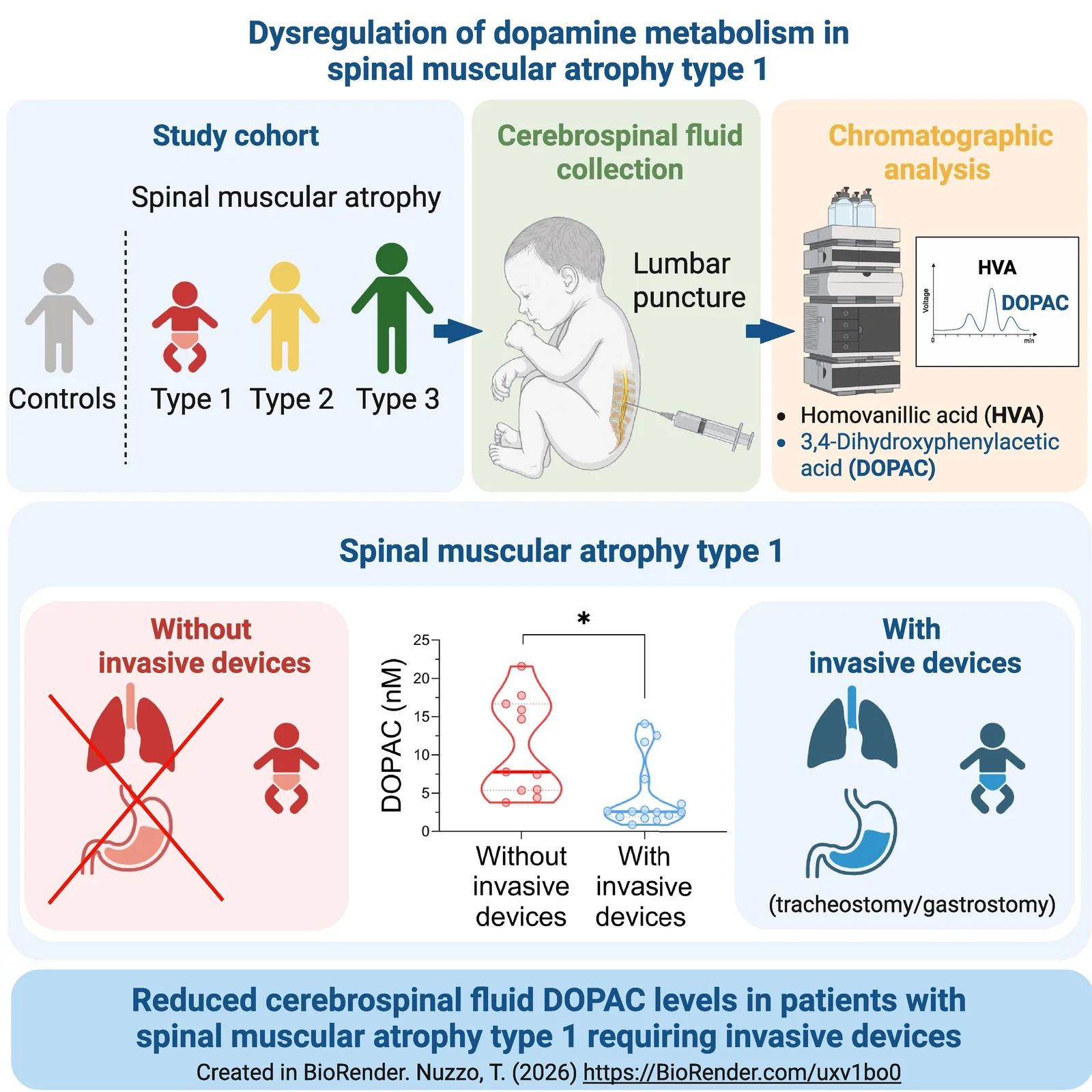

## Introduction

Spinal muscular atrophy (SMA) is a rare, inherited disease characterized by degeneration of lower motor neurons,^1,2^ due to homozygous deletions or mutations in the *survival motor neuron 1* (*SMN1)* gene,^3^ resulting in reduced levels of SMN protein. Clinically, it is classified into subtypes based on age at onset and the highest motor milestone achieved: SMA type 1 (SMA1) represents the most severe early onset form; SMA2 is defined by the ability to sit independently and SMA3 by preserved ambulation, which may be progressively lost over time.^2^

In the last decade, disease-modifying therapies targeting the alternative splicing of the paralogous gene *SMN2* have profoundly altered the natural history of SMA. By increasing exon 7 inclusion in *SMN2* transcripts, these treatments restore the production of full-length functional SMN protein, which is otherwise generated only in limited amounts due to inefficient splicing of *SMN2*. These include Nusinersen (Spinraza), an antisense oligonucleotide administered intrathecally every 4 months (maintenance regimen),^4^ and Risdiplam (Evrysdi), a small molecule administered orally on a daily basis.^5^ More recently, adeno-associated virus-mediated gene replacement therapy has emerged as a mechanistically distinct approach, enabling direct delivery of a functional *SMN1* gene.^6,7^ Irrespective of the specific mechanism of action, these therapeutic approaches increase SMN protein levels and have significantly improved survival and motor outcomes in paediatric patients, particularly those with SMA1, which was uniformly fatal in the pre-treatment era.^8^

Despite these advances, the clinical benefit of SMN-enhancing therapies remains incomplete, especially when treatment is delayed or in patients with a long disease history, probably due to the irreversible loss of motor neuron pools associated with disease progression.^9,10^ More generally, neither therapy has proved capable of completely correcting the symptoms. The incomplete knowledge of the pathogenic effects of SMN deficiency across the central nervous system (CNS) and peripheral organs, along with the absence of reliable biomarkers reflecting the clinical response to SMN induction, represents current limitations to understanding why the current therapies are only partially effective in treating SMA.

SMN is expressed ubiquitously and plays a key role in multiple cellular processes,^11^ including a well-characterized housekeeping function in ribonucleoprotein assembly.^12^ Due to its diverse functions, a deficiency in SMN has been linked to significant metabolic dysregulation, impacting the balance of lipids, glucose, and amino acids in both SMA models and patients.^13-25^ In particular, recent OMIC-based studies have shown that Nusinersen significantly alters the central metabolism of several amino acids, including L-glutamate, L-glutamine, L-serine, L-arginine, branched-chain amino acids and the aromatic amino acids.^23,26-29^ While the mechanistic link between SMN deficiency and amino acid dysregulation remains to be fully elucidated, these findings point to a potential disruption of neurotransmission in SMA. Beyond their primary metabolic roles in protein synthesis and energy homeostasis, these amino acids are directly involved in synaptic transmission as neurotransmitters or serve as their precursors. Accordingly, a growing body of preclinical evidence indicates that synaptic dysfunction represents a central pathogenic feature of SMA, affecting both excitatory and inhibitory circuits within the spinal cord and higher-order CNS networks.^26-28,30-33^

Although much of the earlier work has focused on glutamatergic neurotransmission dysfunction at sensory-motor synapses, increasing attention has recently been directed towards monoaminergic systems. In this context, SMN deficiency has been shown to alter dopamine, norepinephrine and serotonin metabolism in the brain and spinal cord of SMNΔ7 mice, a widely used and well-characterized transgenic model of severe SMA,^34^ especially during the later stages of the disease.^29^ In line with these observations, clinical data indicate that untreated SMA1 patients exhibit elevated cerebrospinal fluid (CSF) levels of aromatic amino acids compared to healthy controls, and that Nusinersen treatment increases norepinephrine levels.^29^ Additionally, a recent *in vivo* study showed that loss of dopaminergic synapses in vulnerable spinal motor neurons causes locomotor and postural deficits in the SMNΔ7 mouse model, and that these deficits were partially rescued by L-dopa and benserazide administration.^35^ Together, earlier results suggest that alterations in neurotransmitter signalling in SMA affect not only spinal motor neuron circuitry but also descending projections from monoaminergic pathways.^30-33,35-37^

Based on these findings linking SMN deficiency to abnormalities in catecholaminergic neurotransmission, in the present work, we investigated dopamine metabolism in SMA patients in a real-world setting. By using high-performance liquid chromatography (HPLC), we quantified the CSF levels of the primary dopamine-related metabolites—3,4-dihydroxyphenylacetic acid (DOPAC) and homovanillic acid (HVA)—in drug-free SMA patients of varying disease severity, compared to healthy controls. As dopamine is highly unstable and prone to rapid oxidation, DOPAC and HVA represent reliable surrogate markers of dopamine metabolism in the CSF and blood.^38,39^ We also investigated the effects of Nusinersen treatment on these metabolites and explored their relationship with clinical measures of disease severity and motor outcome. In addition, serum DOPAC and HVA levels were evaluated in an independent cohort of naïve SMA1 patients and healthy controls. Together, these analyses provide new clinical data on dopamine metabolism in SMA and its potential relationship with disease severity and treatment response.

## Materials and methods

### Patients

This real-world, longitudinal two-centre study included 55 patients with SMA1 (*n* = 26), SMA2 (*n* = 17) or SMA3 (*n* = 12), who were enrolled for CSF analysis and received intrathecal treatment with Nusinersen (12 mg) at the Bambino Gesù Hospital (Rome, Italy) and at the Giannina Gaslini Institute (Genoa, Italy) (Table 1). An independent cohort of drug-naïve SMA1 patients (*n* = 7) was additionally enrolled for serum analyses at the same Italian hospitals (Table 2).

**Table 1:** Demographic and clinical characteristics of treatment-naïve SMA1, SMA2 and SMA3 patients and healthy controls enrolled in the CSF study

	*n* (SMA1:2:3)	Controls (*n* = 6)	SMA1 (*n* = 26)	SMA2 (*n* = 17)	SMA3 (*n* = 12)
Sex, *n* (F:M)	26:17:12	4:2	17:9	11:6	9:3
Age, years	26:17:12	6.5 (3.6–11.2)	3.0 (1.0–4.8)	5.5 (2.0–11.8)	12.8 (4.7–17.1)
*SMN2* copies, *n* (2:3:4)	26:17:12		23:3:0	1:16:0	2:8:2
CHOP-INTEND	26:0:0		14 (4.5–24.7)		
HFMSE	0:17:12			10 (8–14.5)	50 (32.2–55.7)
Gastrostomy, *n* (%)	26:17:12		15 (57.7)	0 (0)	0 (0)
NIV, *n* (%)	26:17:12		11 (42.3)	6 (35.3)	1 (8.3)
Tracheostomy, *n* (%)	26:17:12		8 (30.8)	0 (0)	0 (0)

Continuous variables are presented as median (IQR); categorical variables as absolute frequencies and/or percentages. The number (*n*) of patients is indicated.

CHOP-INTEND, Children’s Hospital of Philadelphia Infant Test of Neuromuscular Disorders; HFMSE, Hammersmith Functional Motor Scale Expanded; NIV, non-invasive ventilation; *SMN2*, Survival Motor Neuron 2.

**Table 2:** Demographic and clinical information of treatment-naïve SMA1 patients and healthy controls enrolled in the serum study

	Controls (*n* = 8)	SMA1 (*n* = 7)
Sex, *n* (F:M)	5:3	5:2
Age, years	0.8 (0.1–4.2)	0.3 (0.1–2)
*SMN2* copies, *n* (2:3:4)		5:2:0
CHOP-INTEND		24 (14–29)
Gastrostomy, *n* (%)		2 (28.6)
NIV, *n* (%)		2 (28.6)
Tracheostomy, *n* (%)		1 (16.7)

Continuous variables are presented as median (IQR); categorical variables as absolute frequencies and/or percentages. The number (*n*) of patients is indicated.

CHOP-INTEND, Children’s Hospital of Philadelphia Infant Test of Neuromuscular Disorders; NIV, non-invasive ventilation; *SMN2*, Survival Motor Neuron 2.

All patients were clinically and genetically diagnosed, and the number of copies of the *SMN2* gene was determined. All SMA1 patients participated in the expanded access programme for compassionate use to patients with the infantile form only, which took place in Italy between November 2016 and November 2017. The overall clinical response to Nusinersen treatment in these patients,^40,41^ as well as in SMA2 and SMA3 patients,^42^ has previously been reported as part of the full Italian cohort.

CSF samples and clinical data were collected according to a standardized protocol at day 0 (T0; baseline) and day 302 (T302; after 5 doses of Nusinersen). Longitudinal analyses were performed in the subgroup of patients for whom both time points were available (*n* = 44). The reduced number of patients evaluated at T302 reflected the unavailability of follow-up CSF samples due to missed visits or clinical constraints.

Two independent cohorts of paediatric control subjects without neurological disorders deriving from the same Italian hospitals were also enrolled to collect CSF (*n* = 6) or serum (*n* = 8), respectively. Demographic and clinical features of patients enrolled in the CSF and serum analyses are reported in Tables 1 and 2, respectively. The study was approved by the local Ethics Committee of the two hospitals (2395_OPBG_2021). Written informed consent was obtained from all participants and/or their legal guardians. All relevant ethical regulations for human research participants were followed.

### Clinical assessments

Patients enrolled in CSF collection were assessed at baseline (T0) and after the fifth infusion of Nusinersen (T302) using the Children’s Hospital of Philadelphia Infant Test of Neuromuscular Disorders (CHOP-INTEND)^43,44^ or Hammersmith Functional Motor Scale Expanded (HFMSE),^45,46^ based on patients’ functional abilities and age. Ventilation requirements, either non-invasive (NIV) or invasive (tracheostomy), and feeding support (gastrostomy) were also collected (Table 1).

At baseline, all SMA1 patients were non-sitters, with CHOP-INTEND scores ranging from 4.5 to 24.7. HFMSE scores ranged from 8 to 14.5 in SMA2 patients and from 32.2 to 55.7 in SMA3 patients. At the start of the treatment, 20 of the 26 SMA1 patients were older than 1 year, with ages ranging from 1 year and 1 month to 7 years and 8 months. Eight patients had a tracheostomy, 11 were under non-invasive ventilation (NIV) for <16 h/day and 7 patients were in spontaneous breathing. Fifteen patients had gastrostomy. The age of the seventeen SMA2 patients ranged from 11 months to 13 years and 3 months at baseline. Six of these patients were under NIV, and none had gastrostomy or tracheostomy. Among the 12 SMA3 patients, one was under NIV for <16 h/day, and none had gastrostomy.

SMA1 patients enrolled in serum collection were assessed at T0 using the CHOP-INTEND^43,44^ score.

All SMA1 patients were non-sitters, with CHOP-INTEND scores ranging from 7 to 40. The age range was from 1 month to 3 years and 7 months. One patient had a tracheostomy, two were under NIV for <16 h/day and four patients were in spontaneous breathing. Two patients had gastrostomy (Table 2).

### Intrathecal treatment with Nusinersen and CSF collection

Intrathecal administration of Nusinersen (12 mg) was performed in a hospital setting, as previously reported.^23^ CSF samples were collected in polypropylene tubes and stored at −80°C until further analysis. Among the 26 SMA1 patients, CSF samples were obtained from all patients at the first injection (T0), and from 17 patients at T302. Among the 17 SMA2 patients, CSF samples were collected from all patients at T0 and from 15 patients at T302. For the 12 SMA3 patients, CSF samples were collected from all patients at both T0 and T302. The sample size was determined by the number of eligible SMA patients treated at the two centres during the study period; no formal sample size calculation was performed due to the exploratory nature of the study. Missing follow-up CSF samples were due to clinical or procedural reasons; no imputation was performed. Loss to follow-up was handled by restricting longitudinal analyses to patients with paired T0–T302 samples. Exclusion criteria included the presence of symptoms or laboratory abnormalities in blood biochemical and haematological parameters suggestive of a systemic inflammatory state, as well as the use of immunosuppressive treatments within the 6 months preceding enrolment.

### HPLC detection

Twenty microlitres of each sample (5 μl of hydrophilic phase from CSF or serum + 15 μl of 0.2 N PCA) were injected without purification into an HPLC equipped with a reverse phase column (LC-18 DB, 15 cm, 5 μm particle size, Supelco, Waters, Milford, MA, USA) and a coulometric detector (ESA, Coulochem II, Bedford, MA, USA) to quantify DOPAC and HVA.

To detect these catabolites, the first electrode of the detector was set at +125 mV (oxidation) and the second at −175 mV (reduction). The composition of the mobile phase was (in mM): 50 NaH_2_PO_4_, 0.1 Na_2_-EDTA, 0.5 *n*-octyl sodium sulphate, 15% (v/v) methanol, pH 3.79394. The composition of the mobile phase was: 120 mM CH_3_-COONa, 100 mM citric acid, 0.3 mM EDTA, 5% (v/v) methanol, pH 4.995. For quantitative determination of each neurotransmitter, calibration curves were run using standards (Sigma-Aldrich, MO, USA). For data acquisition, the software ESA CDS (Euroservice, Ge, Italy) was used. Final values were expressed as nM. All samples were analysed using the same instrumentation, analytical conditions and calibration procedures to minimize measurement bias.

### Statistical analysis

Statistical analyses were performed using SPSS software v.26 (SPSS Inc., Chicago, IL, USA), with the statistical significance level set to *P* ≤ 0.05. Normality distribution was assessed by the Kolmogorov–Smirnov and Shapiro–Wilk tests. Continuous variables were summarized as the median and interquartile range (IQR), while qualitative variables were summarized by absolute or relative frequency. Differences between diagnostic groups were studied by non-parametric Mann–Whitney test or Kruskal–Wallis test followed, if statistically significant, by *post hoc* tests performed by Mann–Whitney test with Bonferroni’s correction. The effect of confounders (sex, age) was evaluated by ANCOVA on natural log-transformed variables. ANCOVA, corrected for sex and age, was also applied to test for differences between patients with and without gastrostomy or tracheostomy.

Differences between dependent groups (T0–T302) were studied by Wilcoxon matched-pairs signed-rank test. Association between continuous variables was evaluated by non-parametric partial correlation, corrected by age as a confounder. The effect of age on motor outcome was further tested through Spearman correlation analysis between the standardized residuals derived from linear regression models (with age entered as the independent variable and DOPAC or ΔCHOP as dependent variables). No sensitivity analyses were performed due to the exploratory nature of the study.

## Results

### Analysis of dopamine-related metabolites in the CSF of SMA patients at baseline and after Nusinersen treatment

We investigated whether SMN deficiency affects the concentrations of dopamine-related metabolites, measuring DOPAC and HVA by HPLC (Fig. 1A) in the CSF of treatment-naïve SMA1 (*n* = 26), SMA2 (*n* = 17) and SMA3 (*n* = 12) patients and healthy controls (*n* = 6), whose clinical and demographic features are detailed in Table 1. Non-parametric Kruskal–Wallis analysis showed comparable CSF concentrations of both DOPAC and HVA across SMA patients and controls (Fig. 1B and C; Table S1). These findings were confirmed by age- and sex-adjusted ANCOVA (Table S1).

**Figure 1:**
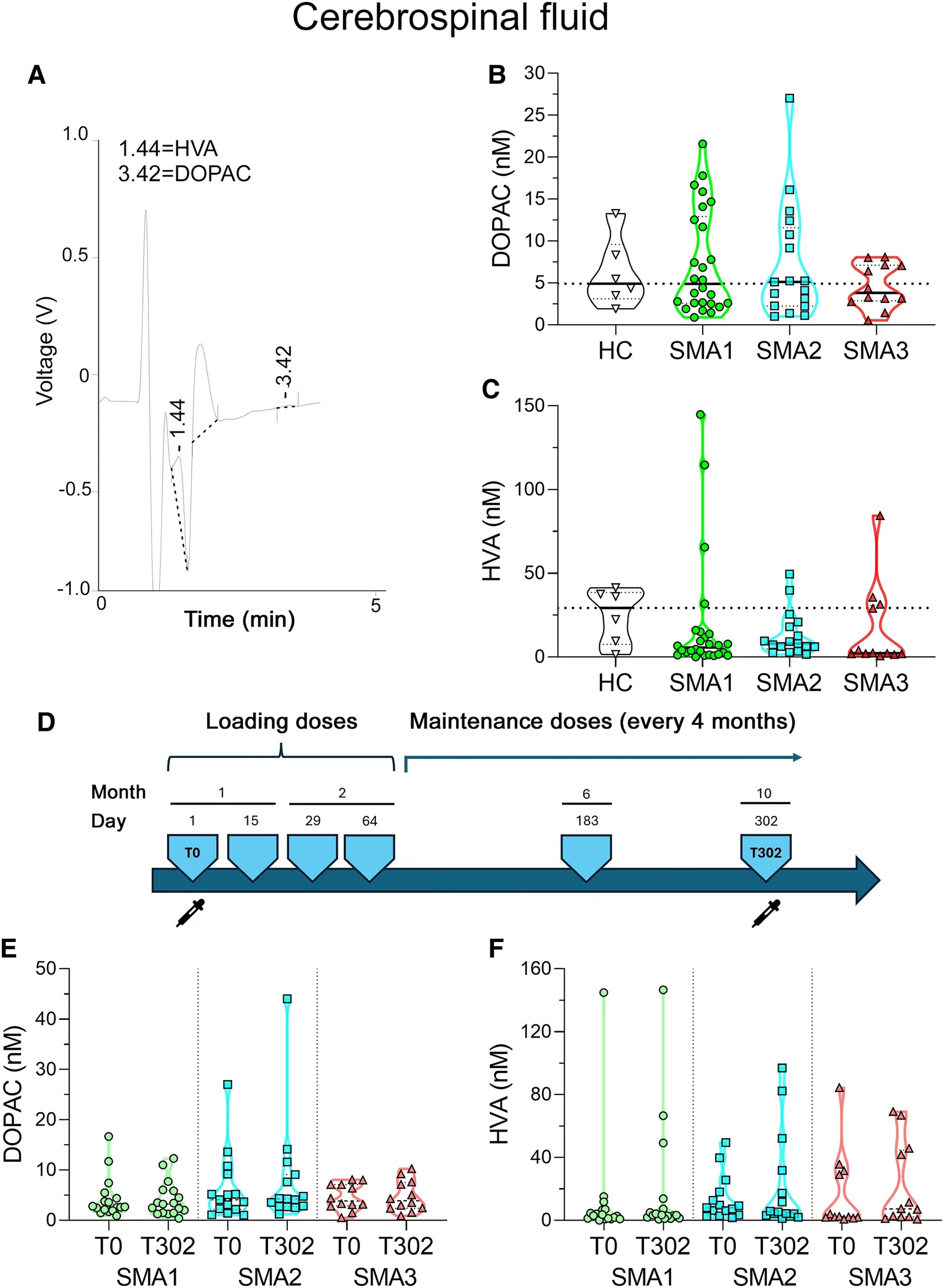
**CSF DOPAC and HVA levels in SMA patients at baseline and after Nusinersen treatment.** (**A**) Representative chromatogram showing DOPAC and HVA peaks in CSF samples from SMA patients. (**B**) DOPAC and (**C**) HVA levels in treatment-naïve SMA1 (*n* = 26), SMA2 (*n* = 17), SMA3 (*n* = 12) patients and healthy controls (HC, *n* = 6). (**D**) Timeline of intrathecal Nusinersen administration (12 mg/dose) and CSF collection. CSF samples were collected at baseline (T0) and at the time of the sixth Nusinersen injection (T302). Longitudinal analysis of (**E**) DOPAC and (**F**) HVA levels in the CSF of SMA1 (*n* = 17), SMA2 (*n* = 15) and SMA3 (*n* = 12) patients at T0 and T302. Statistical analyses were performed using the Kruskal–Wallis test and ANCOVA on ln-transformed values with age as covariate for panels (**B**, **C**), and the Wilcoxon signed-rank test for panels (**E, F**). Data are shown as violin plots representing the median with IQR. Dots represent individual patients’ values.

We then investigated whether Nusinersen administration altered CSF dopamine metabolite levels. No significant differences in DOPAC or HVA levels were observed between T0 (baseline) and T302 (maintenance phase of Nusinersen therapy) in SMA1 (*n* = 17), SMA2 (*n* = 15) and SMA3 (*n* = 12) patients (Fig. 1D–F; Table S2).

### Association of CSF dopamine-related metabolites with disease severity in naïve and Nusinersen-treated SMA patients

We next assessed the association between dopamine-related metabolites and clinical interventions reflecting disease severity, including nutritional and respiratory support. Since gastrostomy and tracheostomy were present exclusively among the most severely affected SMA1 patients in our cohort, subsequent analyses were restricted to this subgroup.

Treatment-naïve SMA1 patients requiring either gastrostomy or tracheostomy (*n* = 15) exhibited significantly lower CSF DOPAC levels compared with those without invasive devices (*n* = 11) [ANCOVA: *F*(1,21) = 8.355, *η*_p_^2^ = 0.285, *P*-value = 0.009; Fig. 2A; Table S3]. SMA1 patients requiring supportive interventions were also significantly older than those without them [median age (IQR): 4.0 (2.9–5.6) versus 0.8 (0.4–2.2) years, respectively; *P* = 0.001]. CSF DOPAC levels also showed a negative correlation with age among SMA1 patients (*ρ* = −0.451, *P* = 0.021; Table S4), consistent with the known age-dependent decline of dopamine catabolites in paediatric populations.^47-49^

**Figure 2:**
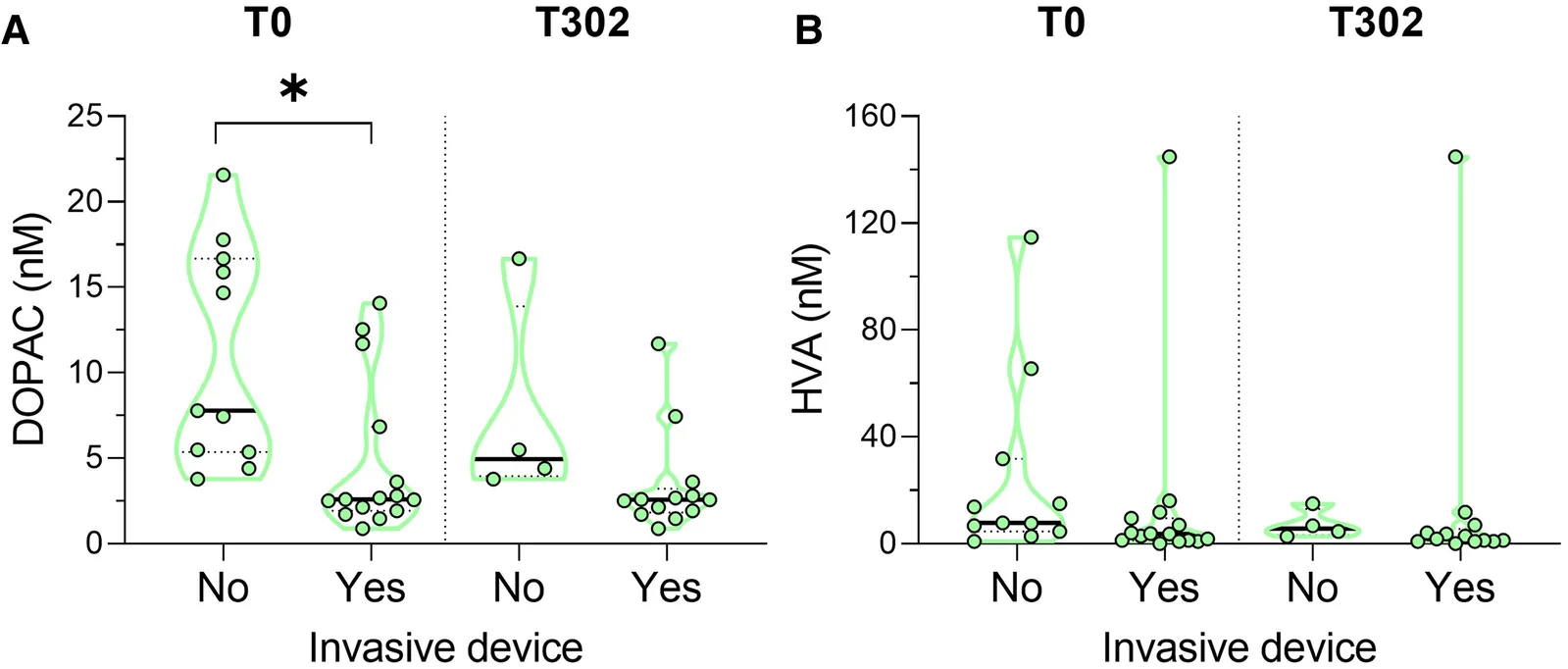
**Longitudinal CSF DOPAC and HVA levels in SMA1 patients stratified by invasive device status.** (**A**) DOPAC and (**B**) HVA levels in SMA1 patients with or without invasive devices (tracheostomy and/or gastrostomy; No, *n* = 11; Yes, *n* = 15) at baseline (T0) and after five doses of Nusinersen (T302). Data are shown as violin plots representing the median with IQR. Dots represent individual values. Statistical analyses were performed using ANCOVA on ln-transformed values with age as covariate. * *P* < 0.05, compared with patients not requiring invasive devices.

Notably, CSF DOPAC alterations were no longer significant after Nusinersen treatment, although a trend towards lower levels was maintained (Fig. 2A; Table S3). In contrast, no significant differences in CSF HVA levels were detected either at baseline or after treatment (Fig. 2B; Table S3).

We then investigated the association between CSF dopamine-related metabolite levels and *SMN2* copy number across the entire SMA cohort. In a separate analysis, we examined their relationship with the requirement for NIV. For this analysis, SMA1 and SMA2 patients were pooled, as only one patient in the SMA3 group required NIV. No significant differences in CSF DOPAC or HVA concentrations emerged between SMA patients with fewer than three copies (*n* = 26) and those with three or more copies (*n* = 29) or between patients with (*n* = 17) and without NIV (*n* = 26) (Table S5).

### Relationship of CSF dopamine-related metabolites with motor function in naïve and Nusinersen-treated SMA patients

We also investigated the correlation between CSF DOPAC or HVA levels and motor function, assessed using CHOP-INTEND or HFMSE scores at baseline and after Nusinersen treatment. Spearman’s correlation analysis revealed no significant associations, except for a positive correlation between baseline DOPAC levels and CHOP-INTEND scores in SMA1 patients at T0 (*n* = 26; *ρ* = 0.531, *P* = 0.005; Table S6). However, after adjustment for age, this association did not retain statistical significance, although a clear positive trend persisted (*ρ* = 0.532, *P* = 0.062; Fig. S1, Table S6). Consistently, baseline DOPAC levels were positively correlated with longitudinal motor improvement following Nusinersen treatment, measured as ΔCHOP-INTEND (T302 – T0) (*n* = 17; *r* = 0.640, *P* = 0.006) (Fig. 3, Table S7). Notably, this correlation remained statistically significant after correction for age (*ρ* = 0.538, *P* = 0.032, Table S7), suggesting that higher baseline DOPAC levels are predictive of enhanced motor improvement after Nusinersen treatment.

**Figure 3:**
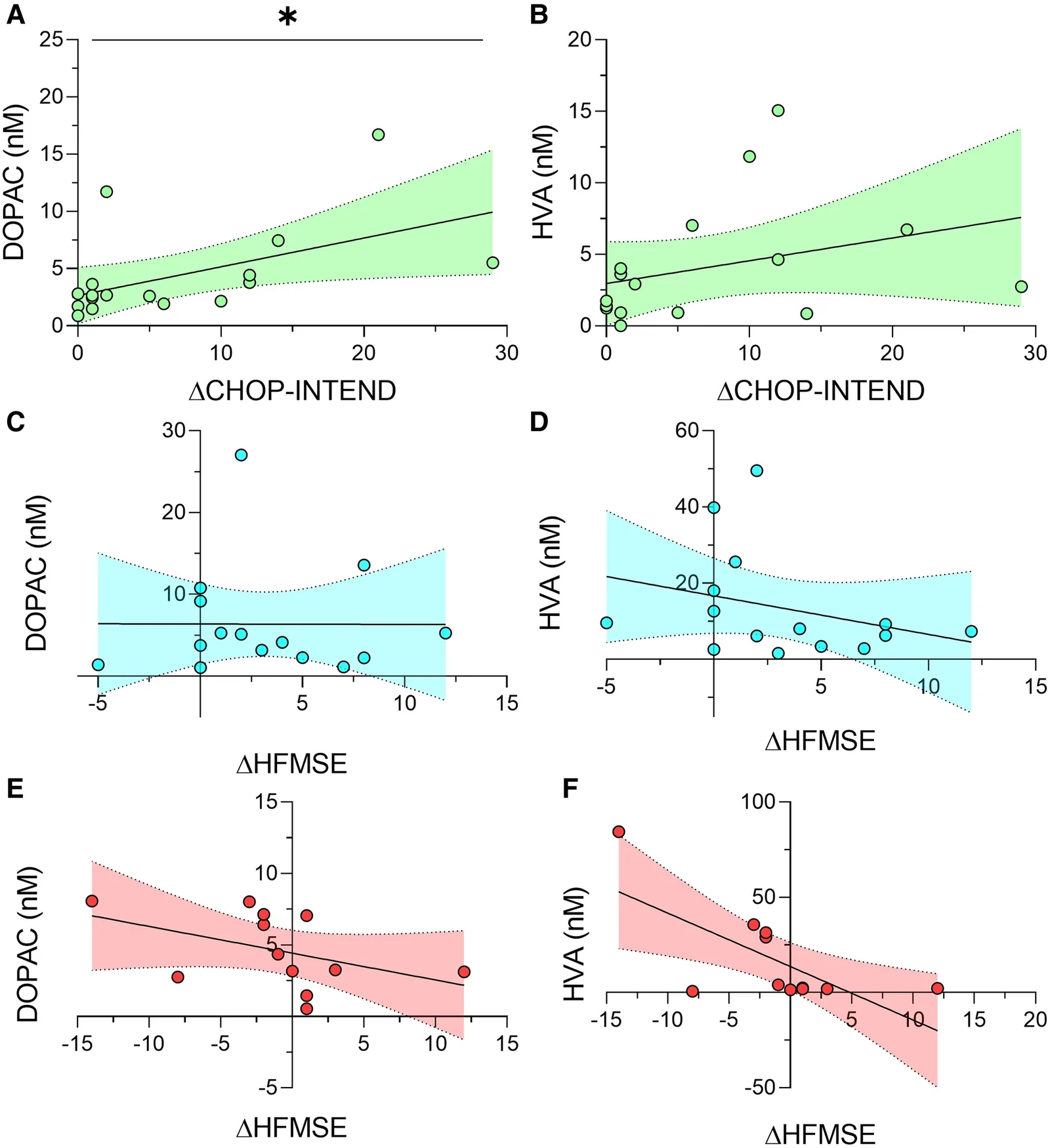
**Age-adjusted correlations between CSF DOPAC or HVA levels and motor improvement following Nusinersen treatment.** Correlations were assessed between baseline CSF (**A, C, E**) DOPAC or (**B, D, F**) HVA levels and (**A, B**) ΔCHOP-INTEND scores in SMA1 patients (*n* = 17) or **C–F**) ΔHFMSE scores in (**C, D**) SMA2 (*n* = 15) and (**E, F**) SMA3 (*n* = 12) patients. Analyses were performed using age-adjusted Spearman correlation. *P < 0.05.

To further evaluate whether the observed associations were influenced by age, we repeated the correlation analyses after removing the effect of age from both DOPAC and motor outcome measures. Specifically, Spearman correlations were performed between the age-adjusted residuals of DOPAC and those of CHOP-INTEND or ΔCHOP-INTEND. No significant correlations were observed between baseline DOPAC levels and CHOP-INTEND scores (*ρ* = 0.162, *P* = 0.428; Fig. S2A), nor between baseline DOPAC levels and ΔCHOP-INTEND (*ρ* = 0.355, *P* = 0.162; Fig. S2B).

### Analysis of dopamine-related metabolites in the serum of SMA1 patients and healthy controls

Finally, we explored circulating dopamine-related metabolites in an independent cohort of treatment-naïve SMA1 patients (*n* = 7) and sex-matched healthy controls (*n* = 8). Clinical and demographic features of this cohort are reported in Table 2.

Age-adjusted ANCOVA showed no significant difference in serum DOPAC levels between SMA1 patients and healthy controls, whereas serum HVA levels were reduced in SMA1 patients [*F*(1,12) = 5.297, *P* = 0.04; Fig. 4, Table S8]. In addition, Spearman’s correlation analysis showed no significant association between serum DOPAC levels and CHOP-INTEND scores (Table S9). Serum HVA levels showed positive correlation with motor function; however, this association did not retain statistical significance after correction for age (Table S9). Interestingly, while CSF alterations were more evident for DOPAC, the strongest peripheral associations involved HVA, pointing to potentially distinct central and peripheral signatures of dopamine metabolism in SMA.

**Figure 4:**
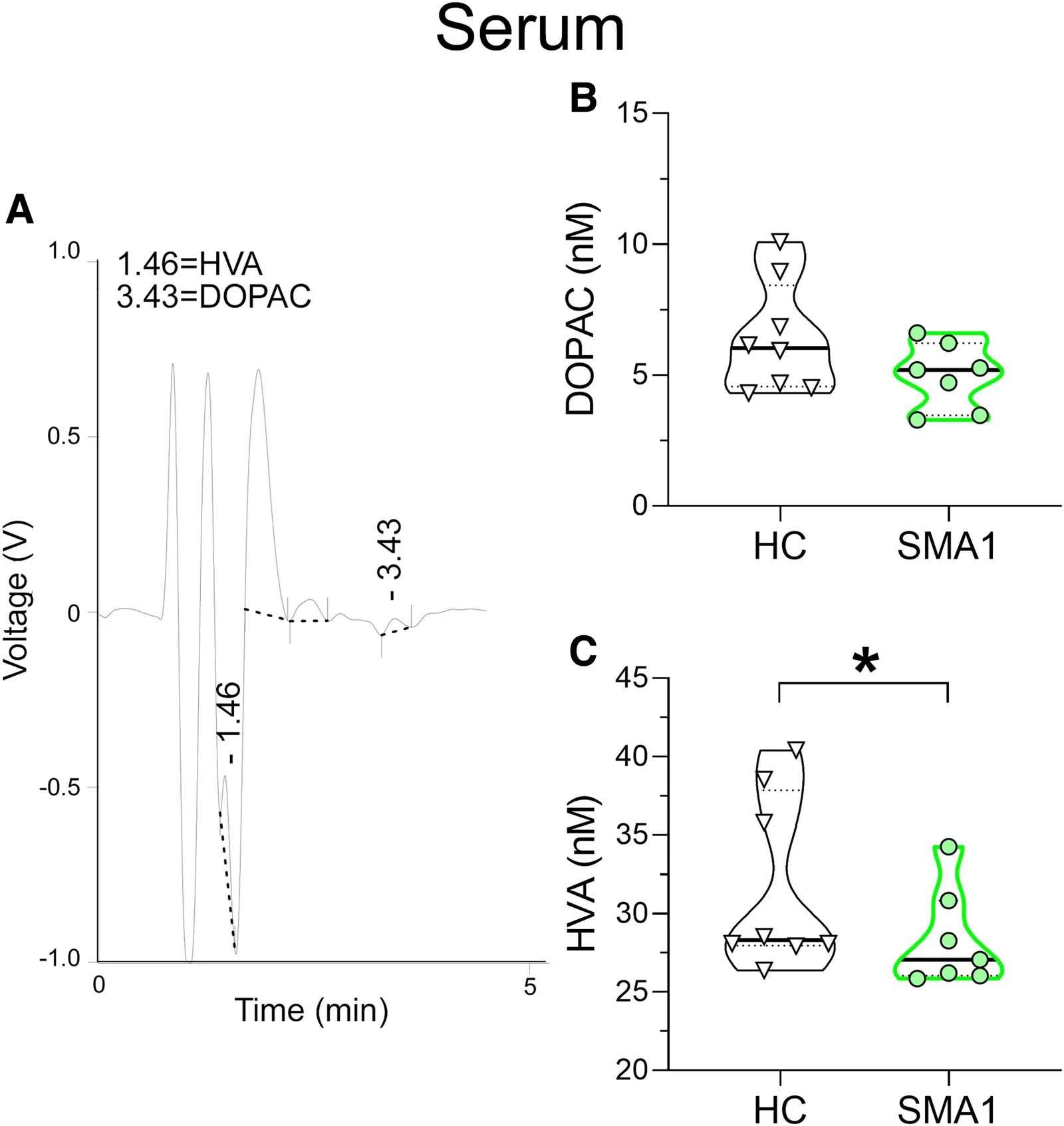
**Serum DOPAC and HVA levels in SMA1 patients and healthy controls.** (**A**) Representative chromatogram showing DOPAC and HVA peaks in serum samples. Serum (**B**) DOPAC and (**C**) HVA levels in treatment-naïve SMA1 patients (*n* = 7) and healthy controls (HC, *n* = 8). Data are shown as violin plots representing the median and IQR. Dots represent individual values. Group comparisons were performed using ANCOVA on ln-transformed values with age as covariate. *P < 0.05.

## Discussion

The CSF levels of dopamine-related catabolites have been extensively investigated in Parkinson’s disease and atypical parkinsonism as biomarkers of nigrostriatal dopaminergic degeneration and disease progression.^50-54^ A hallmark of Parkinson’s disease is the progressive loss of dopaminergic neurons in the substantia nigra, which is considered the main driver of motor symptoms.^55^ Although fatigue, motor and postural impairment are also key clinical features of SMA,^2^ the involvement of central dopaminergic metabolism in this neurodegenerative disease has not been previously explored. In the present study, we addressed this issue by measuring CSF levels of the two main dopamine catabolites, DOPAC and HVA, in SMA patients at baseline and after intrathecal Nusinersen treatment.

We found no significant differences in baseline CSF DOPAC or HVA concentrations across SMA types, indicating the absence of a clear subtype-dependent alteration in central dopamine-related catabolites. Although no significant association with age was detected, a downward trend in both metabolites was observed from SMA1 to SMA3. Given the progressive increase in median age across SMA subtypes, this pattern is broadly consistent with the well-established age-related decline of dopamine metabolism reported in paediatric populations.^47-49,56^ However, the limited number of age-matched healthy controls precludes firm conclusions regarding the contribution of physiological aging to these observations.

A different picture emerged when disease severity was examined within the most affected subgroup. Indeed, in treatment-naïve SMA1 patients, lower CSF DOPAC levels were associated with the presence of gastrostomy or tracheostomy, suggesting a relationship between reduced dopamine metabolism and more severe clinical status. However, it should be acknowledged that the absence of invasive devices does not necessarily reflect a milder clinical condition, as caregiver preferences and clinical decision-making may also influence their use.^57^ Moreover, SMA1 patients requiring these interventions tended to be older than those who did not, consistent with longer disease duration and more advanced disease stage. In line with these observations, age-adjusted correlation analyses showed that lower baseline DOPAC levels were associated with reduced motor improvement following Nusinersen treatment, as indicated by smaller changes in ΔCHOP-INTEND scores. These findings should nevertheless be interpreted cautiously and warrant validation in larger, independent cohorts. Together, these results suggest that altered dopaminergic metabolism accompanies advanced disease stages in SMA1 and may identify patients with more severe motor impairment and reduced functional recovery.

Exploratory analyses performed in an independent cohort of SMA1 patients and sex-matched healthy controls revealed significantly reduced serum HVA levels in patients, while serum DOPAC levels showed a non-significant trend in the same direction. Although exploratory and limited by sample size, these observations suggest that alterations in dopamine-related metabolites may extend beyond the CNS. Interestingly, whereas DOPAC represented the metabolite most consistently associated with disease severity in CSF, HVA showed the clearest peripheral alteration. This apparent discrepancy may reflect tissue-specific regulation of dopamine metabolism, differences in the underlying enzymatic pathways, and contributions from non-neuronal sources, such as the adrenal glands.^58^

The selective downregulation of CSF DOPAC levels observed in SMA1 patients requiring gastrostomy or tracheostomy may reflect greater perturbations of dopaminergic neurotransmission associated with increased clinical burden, rather than a direct effect of these interventions. In this regard, both human and animal studies have shown that chronic stress conditions are associated with reduced dopamine and dopamine-related metabolite levels in CSF and mesolimbic brain regions, respectively.^59-64^ It is therefore plausible that the greater disease burden contributes to the dopaminergic metabolic alterations observed in the most severe SMA1 patients. However, the extent to which these findings reflect disease severity itself, treatment-related factors, or broader systemic stress responses cannot be determined from the present data and warrants further investigation. Additional confounding factors should also be considered. Both increased caloric intake and caloric restriction are known to influence central and peripheral catecholamine levels.^65^ Accordingly, enteral feeding may have altered nutrient composition and energy balance, potentially impacting CNS dopaminergic metabolism in patients treated with gastrostomy. Further preclinical and clinical studies are thus warranted to elucidate the contribution of these factors.

Notably, we previously reported substantial cerebral alterations of dopamine metabolism in late-stage SMNΔ7 mice, including changes in the expression of dopamine-regulating enzymes and increased cerebral dopamine content.^29^ These findings are not necessarily discordant with the present clinical data, as CSF DOPAC reflects extracellular dopamine turnover across multiple brain regions rather than capturing tissue- or circuit-specific dopaminergic alterations.^65^ Differences in disease stage, compensatory adaptations and chronic disease burden may therefore contribute to the distinct neurochemical signatures observed in experimental models and patients. Overall, our findings suggest that CSF DOPAC levels mainly reflect disease stage and clinical severity, rather than the molecular mechanisms directly linking SMN deficiency to altered dopamine metabolism.

Supporting the functional relevance of dopaminergic system dysregulation in SMA, a recent study demonstrated that loss of catecholaminergic synapses from spinal neurons contributes to neurological deficits in SMNΔ7 mice and that these abnormalities can be partially rescued by L-dopa and benserazide treatment.^35^ Notably, the number of TH-positive neurons in the substantia nigra pars compacta, ventral tegmental area^29^ and A11 hypothalamic region^35^ was preserved in SMNΔ7 mice, suggesting that dopaminergic dysfunction arises from neurometabolic and synaptic alterations rather than dopaminergic neuron loss. Together with the present association between lower CSF DOPAC levels and greater disease severity, these findings support dopaminergic pathways as complementary therapeutic targets alongside SMN-enhancing strategies.

Beyond motor control, the dopaminergic system plays a crucial role in cognition, mood and motivation.^65-70^ Emerging evidence indicates that severe SMA1 patients may develop cognitive and social deficits despite early SMN-restoring therapies.^71^ Moreover, recent studies have identified cerebellar Purkinje cell degeneration and altered cerebellar circuit activity in both SMA1 patients and *SMNΔ7* mice, with cerebellar abnormalities contributing to social communication impairments in mouse models.^72^ The potential relevance of dopaminergic mechanisms in these non-motor manifestations is further supported by recent evidence showing that Purkinje cells can synthesize and release dopamine within cerebellar circuits.^73^ Although neuropsychiatric outcomes were not assessed in the present study, the dopaminergic dysregulation observed in advanced SMA may contribute to cognitive and behavioural alterations and deserves further investigation.

Lower CSF concentrations of dopamine-related metabolites have been associated with greater motor dysfunction^53,54^ and loss of dopaminergic terminals in Parkinson’s disease.^52,53,74^ Although the dopaminergic pathways involved in Parkinson’s disease and SMA pathogenesis differ substantially (nigrostriatal^55^ projections in Parkinson’s disease versus A11 hypothalamic projections in SMA^35^), the level of dopamine catabolites may represent informative markers of dopaminergic dysmetabolism and motor impairment across distinct neurodegenerative disorders.

This exploratory study has some limitations. The relatively small sample size of the clinical subgroups limits statistical power and generalizability. Similarly, the small control group and the lack of an independent validation cohort hinder the interpretation of baseline metabolite levels. In addition, the analysis relied primarily on CSF measurements, which reflect a composite signal from multiple CNS regions^75-78^ and may mask localized alterations. Although CSF collection was standardized,^28^ the physiological dynamics of this biofluid preclude region- or circuit-specific mechanistic inferences. Finally, other confounding variables, including enteral nutrition, chronic psychosocial stress and age-related changes in dopamine catabolites during early life,^47-49^ may have influenced the observed neurochemical signatures.

In conclusion, lower CSF DOPAC levels were associated with greater disease severity and poorer motor response in SMA1 patients. Although these findings are exploratory and do not imply causality, they suggest that altered dopaminergic metabolism accompanies advanced disease stages and may contribute to functional impairment in severe SMA. Given the growing recognition of SMA as a multisystem disease,^79^ dopaminergic neurotransmission warrants further investigation as a potential adjunctive therapeutic target alongside SMN-targeted therapeutic approaches.

## Supplementary Material

fcag402_Supplementary_Data

## Data Availability

The data that support the findings of this study are available from the corresponding author upon reasonable request.
