# Supplementary material for: Dysregulation of dopamine metabolism in children with spinal muscular atrophy type 1
Source: Brain Commun. 2026 Oct 29;8(6):fcag402. doi: 10.1093/braincomms/fcag402 (PMC13733384; doi:10.1093/braincomms/fcag402)
Supplement: Supplementary file 1 [file fcag402_supplementary_data.docx]

**Supplementary information for**

**Dysregulation of dopamine metabolism in children with spinal muscular atrophy type 1**

**Short title:** Dopamine metabolism in SMA1

Tommaso Nuzzo^1,2,^*, Barbara Risi^3,4,^*, Valentina Bassareo^5,^*, Adele D’Amico^6^, Alberto Imarisio^7,8^, Antonella Longo^6^, Manolo Carta^5^, Chiara Panicucci^9^, Claudio Bruno^9,10^, Enza Maria Valente^7,8^, Massimiliano Filosto^4,11^, Enrico Bertini^6^, Francesco Errico^1,12,@^, Alessandro Usiello^1,2,@^

1 Laboratory of Translational Neuroscience, Ceinge Biotecnologie Avanzate "Franco Salvatore", 80145, Naples, Italy

2 Department of Environmental, Biological and Pharmaceutical Science and Technologies, Università degli Studi della Campania "Luigi Vanvitelli", 81100, Caserta, Italy

3 Department of Molecular and Translational Medicine, University of Brescia, Brescia, Italy

4 NeMO-Brescia Clinical Center for Neuromuscular Diseases, Brescia, Italy

5 Department of Biomedical Sciences, University of Cagliari, 09042, Monserrato, Italy

6 Unit of Neuromuscular and Neurodegenerative Disorders, Dept. Neurosciences, Bambino Gesù Children's Hospital IRCCS, 00163, Rome, Italy

7 Department of Molecular Medicine, University of Pavia, 27100, Pavia, Italy

8 Neurogenetics Research Centre, IRCCS Mondino Foundation, 27100, Pavia, Italy

9 Center of Translational and Experimental Myology, IRCCS Istituto Giannina Gaslini, Genoa, Italy

10 Department of Neuroscience, Rehabilitation, Ophthalmology, Genetics, Maternal, and Child Health - DINOGMI, University of Genova, Genoa, Italy

11 Department of Clinical and Experimental Sciences, University of Brescia, Brescia, Italy

12 Department of Agricultural Sciences, University of Naples “Federico II”, 80055, Portici, Italy

* Tommaso Nuzzo, Barbara Risi and Valentina Bassareo contributed equally to this work.

@ Corresponding Authors:

Francesco Errico, Department of Agricultural Sciences, University of Naples “Federico II”, Via Università, 100 – 80055 – Portici (NA), Italy; e-mail: francesco.errico@ceinge.unina.it;

Alessandro Usiello, Department of Environmental, Biological and Pharmaceutical Sciences and Technologies, University of Campania “Luigi Vanvitelli”, Via Vivaldi, 43 – 81100 – Caserta, Italy; e-mail: alessandro.usiello@unicampania.it.

**Supplementary Table 1.** CSF DOPAC and HVA levels in treatment-naïve SMA patients.

|  | **Controls**  **(n=6)** | **SMA1**  **(n=26)** | **SMA2**  **(n=17)** | **SMA3**  **(n=12)** | ***p* value^1^** | ***p* value^2^** |
| --- | --- | --- | --- | --- | --- | --- |
| **DOPAC (nM)** | 4.9 (3.1-9.6) | 4.9 (0.9-21.6) | 5.1 (1.0-27.0) | 3.8 (0.5-8.1) | 0.864 | 0.962 |
| **HVA (nM)** | 29.3 (7.6-38.5) | 5.7 (1.6-14.1) | 8.0 (4.8-19.5) | 2.3 (1.9-30.9) | 0.188 | 0.133 |

Data are expressed as median (interquartile range, IQR). ^1^Group comparisons were performed using a non-parametric Kruskal-Wallis test. ^2^ANCOVA was conducted on ln-transformed values (due to non-normally distributed residuals) with age as a covariate. The number (n) of patients is indicated. Abbreviations: 3,4-dihydroxyphenylacetic acid (DOPAC), homovanillic acid (HVA).

**Supplementary Table 2.** Longitudinal changes in CSF DOPAC and HVA levels in Nusinersen-treated SMA patients between T0 and T302.

| **Metabolite** | **SMA1 (n=17)** | **SMA2 (n=15)** | **SMA3 (n=12)** |
| --- | --- | --- | --- |
|  | **T0 vs T302** | **T0 vs T302** | **T0 vs T302** |
| **DOPAC (nM)** | 2.6 vs 3.2 (*p* = 0.981) | 4.1 vs 4.3 (*p* = 0.173) | 3.8 vs 3.9 (*p* = 0.583) |
| **HVA (nM)** | 2.9 vs 3.3 (*p* = 0.687) | 8.0 vs 5.4 (*p* = 0.865) | 2.3 vs 7.2 (*p* = 0.209) |

Data are expressed as median. *p* values derive from non-parametric Wilcoxon test. The number (n) of patients is indicated. Abbreviation: 3,4-dihydroxyphenylacetic acid (DOPAC), homovanillic acid (HVA).

**Supplementary Table 3.** Basal and post-treatment CSF DOPAC and HVA levels in SMA1 patients, stratified by the presence of any device between gastrostomy or tracheostomy.

|  | **T0** | | | | **T302** | | | |
| --- | --- | --- | --- | --- | --- | --- | --- | --- |
| **Metabolite** | **Invasive device** | | ***F (1,21)*** | ***p value*** | **Invasive device** | | ***F (1,13)*** | ***p value*** |
|  | **No (n=11)** | **Yes (n=15)** |  |  | **No (n=4)** | **Yes (n=13)** |  |  |
| **DOPAC (nM)** | 7.8 (5.4-16.7) | 2.6 (1.9-6.8) | **8.355** | **0.009** | 4.0 (2.0-5.5) | 2.5 (1.4-6.9) | 0.691 | 0.421 |
| **HVA (nM)** | 7.8 (4.6-31.8) | 3.6 (1.2-9.5) | 3.280 | 0.084 | 4.0 (1-6-111.2) | 3.3 (1.7-10.6) | 0.339 | 0.57 |

Data are expressed as median (interquartile range, IQR). *p* values derive from ANCOVA conducted on ln-transformed values with age as covariate. At T0, ANCOVA was conducted between HC, SMA1 without device and with device; at T302, ANCOVA was conducted between SMA1 without and with device. The number (n) of patients is indicated. Abbreviation: 3,4-dihydroxyphenylacetic acid (DOPAC); healthy controls (HC); homovanillic acid (HVA).

**Supplementary Table 4.** Correlation between CSF DOPAC or HVA levels and age in treatment-naïve SMA patients.

| **Parameter** | **Patients** | **DOPAC (nM)** | **HVA (nM)** |
| --- | --- | --- | --- |
| **Age (years)** | **SMA1-2-3 (n=55)** | *r=*-0.196 | *r=*0.032 |
|  |  | *p*=0.152 | *p*=0.818 |
|  | **SMA1 (n=26)** | ***r=*-0.451** | *r=*-0.198 |
|  |  | ***p*=0.021** | *p*=0.332 |
|  | **SMA2 (n=17)** | *r=*0.040 | *r=*0.256 |
|  |  | *p*=0.877 | *p*=0.321 |
|  | **SMA3 (n=12)** | *r=*-0.042 | *r=*0.098 |
|  |  | *p*=0.897 | *p*=0.762 |

Non-parametric Spearman’s rho coefficients (*r*) and related *p* values (*p*) are indicated. Significant *p* values are shown in bold. The number (n) of patients is indicated. Abbreviations: 3,4-dihydroxyphenylacetic acid (DOPAC), homovanillic acid (HVA), total cohort (tot).

**Supplementary Table 5.** Basal and post-treatment CSF DOPAC and HVA levels in SMA patients, stratified by SMN2 copy number or the presence of non-invasive ventilation.

|  | **T0** | | | **T302** | | |
| --- | --- | --- | --- | --- | --- | --- |
| **Metabolite** | **SMN2 cn  (SMA 1-2-3)** | | ***p value*** | **SMN2 cn  (SMA 1-2-3)** | | ***p value*** |
|  | **< 3 (n=26)** | **≥ 3 (n=29)** |  | **< 3 (n=18)** | **≥ 3 (n=26)** |  |
| **DOPAC (nM)** | 3.7 (2.4-12.4) | 5.1 (2.9-8.0) | 0.249 | 3.3 (1.9-5.9) | 4.2 (2.6-8.0) | 0.188 |
| **HVA (nM)** | 3.9 (1.6-12.3) | 7.7 (2.7-27.4) | 0.281 | 3.3 (1.8-7.3) | 6.4 (2.6-47.3) | 0.422 |
|  |  |  |  |  |  |  |
| **Metabolite** | **NIV (SMA1-2)** | | ***p value*** | **NIV (SMA1-2)** | | ***p value*** |
|  | **No (n=26)** | **Yes (n=17)** |  | **No (n=19)** | **Yes (n=13)** |  |
| **DOPAC (nM)** | 4.3 (2.2-16.3) | 5.2 (2.5-12.9) | 0.334 | 3.6 (2.0-7.7) | 3.2 (2.4-6.7) | 0.813 |
| **HVA (nM)** | 6.6 (2.5-9.9) | 9.6 (3.7-28.7) | 0.151 | 3.3 (2.0-13.8) | 4.3 (3.8-24.5) | 0.263 |

Data are expressed as median (interquartile range, IQR). *p* values derive from ANCOVA conducted on ln-transformed values with age as covariate. The number (n) of patients is indicated. Abbreviation: 3,4-dihydroxyphenylacetic acid (DOPAC), homovanillic acid (HVA), non-invasive ventilation (NIV), SMN2 copy number (SMN2 cn).

**Supplementary Table 6.** Correlation between CSF DOPAC or HVA levels and functional motor scales of SMA patients before and after Nusinersen treatment.

| **Functional motor scale** | **Patients** | **Therapy phase** | **DOPAC (nM)** | **HVA (nM)** |
| --- | --- | --- | --- | --- |
| **CHOP-INTEND** | **SMA1** | **T0** | ***r=*0.531** | *r=*0.297 |
|  |  |  | ***p=0.005^a^*** | *p=0.141* |
|  |  |  | n=26 | n=26 |
|  |  | **T302** | *r=*0.264 | *r=*0.040 |
|  |  |  | *p=0.306* | *p=0.877* |
|  |  |  | n=17 | n=17 |
| **HFMSE** | **SMA2** | **T0** | *r=*0.080 | *r=*-0.177 |
|  |  |  | *p=0.758* | *p=0.496* |
|  |  |  | n=17 | n=17 |
|  |  | **T302** | *r=*-0.034 | *r=*-0.500 |
|  |  |  | *p=0.904* | *p=0.058* |
|  |  |  | n=15 | n=15 |
|  | **SMA3** | **T0** | *r=*0.120 | *r=*0.380 |
|  |  |  | *p=0.715* | *p=0.223* |
|  |  |  | n=12 | n=12 |
|  |  | **T302** | *r=*0.000 | *r=*0.042 |
|  |  |  | *p=>0.999* | *p=0.897* |
|  |  |  | n=12 | n=12 |

Non-parametric Spearman’s rho coefficients (r) and related *p* values (*p*) are indicated. Significant *p* values are shown in bold. ^a^ The *p* value does not survive after correction for age (r=0.532, *p*=0.062). Abbreviations: Children’s Hospital of Philadelphia Infant Test of Neuromuscular Disorders (CHOP-INTEND), Hammersmith Functional Motor Scale Expanded (HFMSE), 3,4-dihydroxyphenylacetic acid (DOPAC), homovanillic acid (HVA).

**Supplementary Table 7.** Correlation between baseline CSF DOPAC or HVA levels and changes in functional motor scales in SMA patients after treatment with Nusinersen.

| **Functional motor scale** | **Patients** | **DOPAC (nM)** | **HVA (nM)** |
| --- | --- | --- | --- |
| **ΔCHOP-INTEND** | **SMA1 (n=17)** | ***r=*0.640** | *r=0.350* |
|  |  | ***p*=0.006^a^** | *p*=0.169 |
| **ΔHFMSE** | **SMA2 (n=15)** | *r=0.054* | *r=-0.394* |
|  |  | *p*=0.848 | *p*=0.147 |
|  | **SMA3 (n=12)** | *r=-0.536* | *r=-0.434* |
|  |  | *p*=0.072 | *p*=0.159 |

Non-parametric Spearman’s rho coefficients (*r*) and related *p* values (*p*) are indicated. Significant *p* values are shown in bold. ^a^ The *p* value survives after correction for age (r*=*0.538*, p*=0.032). Abbreviations: Delta (Δ) [T302-T0], Children’s Hospital of Philadelphia Infant Test of Neuromuscular Disorders (CHOP-INTEND), Hammersmith Functional Motor Scale Expanded (HFMSE), 3,4-dihydroxyphenylacetic acid (DOPAC), homovanillic acid (HVA).

**Supplementary Table 8.** Serum levels of DOPAC and HVA compared between naive SMA1 patients and age-matched healthy controls.

| **Metabolite** | **Controls (n=8)** | | | **SMA1 (n=7)** | | | **Mann-Whitney** | **ANCOVA^a^** | |
| --- | --- | --- | --- | --- | --- | --- | --- | --- | --- |
|  | Median | IQR | | Median | IQR | |  | F_(1;12)_ | *p* value |
| **DOPAC (nM)** | 6 | 4.6 | 8.4 | 5.2 | 3.5 | 6.2 | 0.298 | 2.434 | 0.145 |
| **HVA (nM)** | 28.3 | 28 | 37.9 | 27.1 | 26.0 | 30.8 | 0.132 | 5.297 | **0.04** |
| ^a^ Adjusted for age on natural log-transformed data. Statistically significant *p* values are shown in bold. | | | | | | | | | |

| **Supplementary Table 9**. Correlation of serum DOPAC or HVA levels with CHOP-INTEND scores in treatment-naïve SMA1 patients | | | | | | | | |
| --- | --- | --- | --- | --- | --- | --- | --- | --- |
| **Patients** | **Functional motor scale** | **DOPAC (nM)** | **HVA (nM)** |  |  |  | |  |
| **SMA1 (n=7)** | **CHOP- INTEND** | *r= 0.393* | ***r=0.786*** |  |  |  | |  |
|  |  | *p= 0.383* | ***p=0.036^a^*** |  |  |  | |  |
| Non-parametric Spearman’s rho coefficients (*r*) and related *p* values (*p*) are indicated. Significant *p* values are shown in bold. ^a^ The p value does not survive after correction for age (*r=0.246, p=0.638*). The number (n) of patients is indicated. Abbreviations: Children’s Hospital of Philadelphia Infant Test of Neuromuscular Disorders (CHOP-INTEND), 3,4-dihydroxyphenylacetic acid (DOPAC), homovanillic acid (HVA). | | | | | | |  | |

**Supplementary Figure 1. Age-adjusted partial correlations between baseline CSF DOPAC or HVA levels and functional motor scales in naïve and Nusinersen-treated SMA patients.** Partial correlation analyses (corrected for age) between CSF levels of 3,4-dihydroxyphenylacetic acid (DOPAC) or homovanillic acid (HVA) with motor functions are shown for SMA1, SMA2 and SMA3 patients at baseline (T0) and after Nusinersen treatment (T302). Motor function was evaluated using CHOP-INTEND in SMA1 patients and HFMSE in SMA2 and SMA3 patients.

**a	b**

**Supplementary Figure 2**. Spearman correlation between the standardized residuals of baseline DOPAC, baseline CHOP-INTEND or ΔCHOP-INTEND (in treated SMA1 patients) respectively, after regressing out the effect of age. Correlation between DOPAC and CHOP-INTEND in SMA1 patients (n=26) (a) or ΔCHOP-INTEND in treated SMA1 patients (n=17) (b).
